# Functional and structural comparison of visual lateralization in birds – similar but still different

**DOI:** 10.3389/fpsyg.2014.00206

**Published:** 2014-03-25

**Authors:** Martina Manns, Felix Ströckens

**Affiliations:** Department of Biopsychology, Institute of Cognitive Neuroscience, Faculty of Psychology, Ruhr-University BochumBochum, Germany

**Keywords:** cerebral lateralization, visual system, hemispheric strategy, local-global analysis, social recognition, spatial orientation, avian

## Abstract

Vertebrate brains display physiological and anatomical left-right differences, which are related to hemispheric dominances for specific functions. Functional lateralizations likely rely on structural left-right differences in intra- and interhemispheric connectivity patterns that develop in tight gene-environment interactions. The visual systems of chickens and pigeons show that asymmetrical light stimulation during ontogeny induces a dominance of the left hemisphere for visuomotor control that is paralleled by projection asymmetries within the ascending visual pathways. But structural asymmetries vary essentially between both species concerning the affected pathway (thalamo- vs. tectofugal system), constancy of effects (transient vs. permanent), and the hemisphere receiving stronger bilateral input (right vs. left). These discrepancies suggest that at least two aspects of visual processes are influenced by asymmetric light stimulation: (1) visuomotor dominance develops within the ontogenetically stronger stimulated hemisphere but not necessarily in the one receiving stronger bottom-up input. As a secondary consequence of asymmetrical light experience, lateralized top-down mechanisms play a critical role in the emergence of hemispheric dominance. (2) Ontogenetic light experiences may affect the dominant use of left- and right-hemispheric strategies. Evidences from social and spatial cognition tasks indicate that chickens rely more on a right-hemispheric global strategy whereas pigeons display a dominance of the left hemisphere. Thus, behavioral asymmetries are linked to a stronger bilateral input to the right hemisphere in chickens but to the left one in pigeons. The degree of bilateral visual input may determine the dominant visual processing strategy when redundant encoding is possible. This analysis supports that environmental stimulation affects the balance between hemispheric-specific processing by lateralized interactions of bottom-up and top-down systems.

## GENERAL CEREBRAL ASYMMETRIES IN VERTEBRATES

In contrast to original views, cerebral lateralization is a widespread phenomenon in the animal kingdom. Functional and structural differences between left and right brain sides are in no way exclusive for humans but can be found in other vertebrates and even in invertebrates (Vallortigara et al., 1999; Halpern et al., 2005; Vallortigara and Rogers, 2005; Corballis, 2009; Concha et al., 2012; Ocklenburg and Güntürkün, 2012). A widespread functional lateralization is for example the preferential limb use for specific tasks. In mammals, obviously humans show strong hand preferences (Corballis, 2009) but also chimpanzees (Hopkins et al., 2011), mice (Collins, 1975), bats (Zucca et al., 2010) and wallabies (Giljov et al., 2012) show significant side preferences when using their limbs. Furthermore, species of the avian and amphibian class like parrots (Brown and Magat, 2011), chickens (Rogers and Workman, 1993), and toads (Bisazza et al., 1996) show dominance for using one limb on a given task. Strength of lateralization and preferred side differ between species and are in some cases dependent on environmental factors (for an overview, see Ströckens et al., 2013a). Beside limb preference, conspecific vocalization (e.g., language in humans) seems to be broadly lateralized in vertebrates. Most humans show a dominance of the left hemisphere for the production and perception of language (Flöel et al., 2005; Bethmann et al., 2007). Hemispheric dominance for processing conspecific vocalization can also be found in chimpanzees (Taglialatela et al., 2008), sea lions (Böye et al., 2005), dogs (Siniscalchi et al., 2008), or Zebra, and Bengalese finches (Okanoya et al., 2001; Poirier et al., 2009). Interestingly, mammalian species show in all known cases dominance of the left hemisphere for conspecific vocalization while avian species vary in the predominantly used side (for review, see Ocklenburg et al., 2013a). In different species like humans, sheep, or chicken, the right hemisphere is dominant for aspects of social cognition (Brancucci et al., 2009; Corballis, 2009; Daisley et al., 2009; Rosa Salva et al., 2012) as well as spatial processing (Tommasi and Vallortigara, 2001; Vogel et al., 2003; Diekamp et al., 2005; Chiandetti, 2011).

Such hemispheric specializations might be related to differences in hemispheric processing style. Several authors have tried to classify general lateralization patterns and to associate them with hemispheric-specific processing strategies. According to these models, the left hemisphere prefers a serial, or categorical processing style relying on local or high-frequency aspects of stimuli, while the right hemisphere favors parallel or configural processing, encoding global or low-frequency information (e.g., Dien, 2008). For instance, the left-hemispheric dominance for language processing may follow from a left-hemispheric advantage in encoding rapid frequency transitions (Tervaniemi and Hugdahl, 2003). There is evidence that a general dichotomy in encoding information is shared by different vertebrate species and hence, has an evolutionary origin (Vallortigara and Rogers, 2005; Yamazaki et al., 2007; Corballis, 2009; McGilchrist, 2010; Concha et al., 2012).

## THE PUZZLE OF NATURE-NURTURE INTERACTIONS IN GENERATING A LATERALIZED BRAIN

Nevertheless, it is completely unclear how opposed encoding strategies are generated during ontogeny. Similarities between different species and the presence of an asymmetry pattern at the population level suggest a determination by genotypic factors. On the other hand, a high degree of plasticity indicates that envirotypic factors have a strong impact onto the mature lateralization pattern. Biased environmental stimulation, for example, affects hemispheric dominances and how the hemispheres interact to establish and maintain a lateralized functional organization for optimal cognition (Manns, 2006; Concha et al., 2012; Bishop, 2013; Hervé et al., 2013). Further envirotypic factors like hormones or cultural influences can also play a role in the formation of brain asymmetries (Laland, 2008; Schaafsma et al., 2009; Lust et al., 2011). Moreover, geno- and envirotypic effects may converge onto epigenetic processes, like DNA methylation, that ultimately determine lateralization patterns (Poole and Hobert, 2006; Hervé et al., 2013).

Functional asymmetries presumably rely on structural left-right differences in intra- and interhemispheric connectivity patterns (Ocklenburg and Güntürkün, 2012; Hervé et al., 2013; Ocklenburg et al., 2013b) that develop in a tight interplay between geno- and envirotypic factors. Principle differences in the mode of hemispheric-specific processing should be based on variances in the neuronal organization of the left and right brain sides. For example, differences in the neuronal organization of Brodmann area 22 predispose the left hemisphere for speech processing (Galuske et al., 2000). In the human brain, there are gross morphological asymmetries like a leftward asymmetry in planum temporale (for review, see Amunts, 2010) that appear very early during development (Chi et al., 1977). In right-handers, the planum temporale asymmetry is directly related to the left-hemispheric dominance for language processing. Accordingly, they may represent a suitable indicator of cerebral asymmetries. In sinistrals, however, this asymmetry is less pronounced (Geschwind et al., 2002; Foundas et al., 2002; Greve et al., 2013; Meyer et al., 2013). Moreover, pre- and postnatal events can affect asymmetry during development of the planum temporale and disrupt twin concordance (Steinmetz et al., 1995; Eckert et al., 2002). Dissociation between gross morphological and functional asymmetries suggests that they do not reflect left-right differences in the fine structure of neuronal circuits. Recent studies therefore underline the relevance of microstructural differences in human cortical hemispheres that range from dendritic tree features and neuronal cell size up to differences in white matter organization (Stephan et al., 2007; Ocklenburg et al., 2013b).

The microstructural organization of local networks, as well as their afferent and efferent connections, develops in close interactions with envirotypic factors. For more than 50 years, it is known that sensory experience is a critical factor for the activity-dependent fine tuning of neuronal systems (Hubel and Wiesel, 1959; Wong and Ghosh, 2002; West and Greenberg, 2011). Therefore, biased sensory experience can induce subtle differences between the neuronal organization of the left and right brain side, which in turn determine the mature functional lateralization pattern. A neuronal network that is better adjusted to specific processing may enable one hemisphere (a) to adopt dominance for a specific function, (b) to analyze stimuli according to a preferential processing strategy, or (c) to exert dominance in case of conflicts between the hemispheres. It is still under debate, which effects are critical for the establishment of a lateralized functional brain organization (e.g., Bloom and Hynd, 2005; Hervé et al., 2013).

A differentiation between these possibilities requires animal models, which allow modulations of the lateralization pattern by manipulating the action of specific envirotypic factors. The visual system of birds, like chickens or pigeons, is a well suited model for such kind of experiments. In both species, behavioral asymmetries can be associated with morphological left-right differences of the visual pathways at the individual as well as the population level. Critical aspects of these asymmetries depend on unbalanced light stimulation during development (e.g., Vallortigara and Rogers, 2005; Manns, 2006; Rogers et al., 2007; Güntürkün and Manns, 2010). This supports that lateralization is generated within the scope of ontogenetic plasticity and suggests causal relations between structural and functional asymmetries. Although at first glance quite similar, the two avian models display profound differences in the functional and structural outcome that is based on the asymmetrical visual experience. These differences shed light on the interrelations between structural and functional asymmetries that we want to discuss in the following sections. To this end, we start with a short description of avian visual lateralizations and their development followed by a deeper analysis of differences between chickens and pigeons.

## THE LATERALIZED ORGANIZATION OF THE AVIAN VISUAL SYSTEM – A MODEL TO RESOLVE THE PUZZLE

The visual system of birds is lateralized with a pattern that is similar to humans. The left hemisphere dominates the discrimination of small optic details, rule learning, or categorization of visual stimuli. The right hemisphere on the contrary, is in charge of spatial attention and aspects of social cognition (Daisley et al., 2009; Manns and Güntürkün, 2009). These hemispheric specializations can be easily tested just by temporarily occluding one eye with an eye cap, i.e., by monocular testing, since the optic nerves cross virtually completely in birds. Accordingly, information from the left eye is primarily directed to the right brain side and vice versa.

**FIGURE 1:**
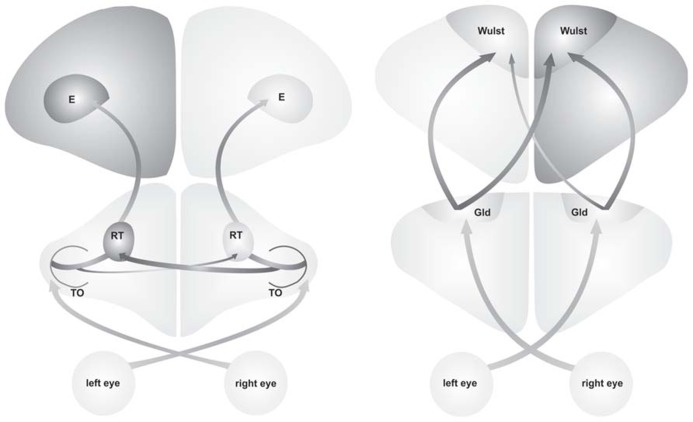
**Visual processing in the tectofugal pathway of pigeons (left side) and the thalamofugal pathway of juvenile chickens (right side) is asymmetrically organized.** In pigeons the projection from the right tectum opticum (TO) to the left nucleus rotundus (RT) are stronger than projection from the right TO to the left RT. In chickens the contralateral projections from left nucleus geniculatus lateralis pars dorsalis (Gld) to the right visual Wulst are stronger than projections from the right Gld to the left visual Wulst. These projection asymmetries lead to higher bilateral input to the left hemisphere in pigeons and to the right hemisphere in pigeons (indicated by darker coloring). These anatomical asymmetries lead to a lateralized behavior in each species (E, entopallium).

Behavioral asymmetries are accompanied by anatomical left-right differences within the ascending visual pathways. In both, pigeons and chickens, structural asymmetries are mainly represented by a difference in projection strength between the two hemispheres. This projection asymmetry corroborates the idea that differences in anatomical connectivity constitute the critical structural substrate of functional asymmetries between the hemispheres (Stephan et al., 2007). But in each species, different visual systems are affected (**Figure 1**). In pigeons, the tectofugal pathway (corresponding to the extrageniculate pathway in mammals) is lateralized, with soma size asymmetries of mesencephalic and diencephalic neurons indicating left-right differences in the complexity of their neuronal connections (Güntürkün, 1997; Manns and Güntürkün, 1999a, 2003; Freund et al., 2008). Moreover, projections of the right optic tectum to the contralateral nucleus rotundus are stronger than the projections of the left tectum to the right rotundus. Since the number of ipsilaterally ascending fibers does not differ between sides, the asymmetry of the contralateral projections effectively increases the total tectal input on the left rotundus (Güntürkün et al., 1998). Thus, it is the left hemisphere that receives a more complete representation of information from both visual hemifields (Valencia-Alfonso et al., 2009). The second major visual pathway aside from the tectofugal, the thalamofugal pathway (corresponding to the geniculo-cortical pathway in mammals), is not lateralized in pigeons, neither in young nor adult birds (Ströckens et al., 2013b). In chickens, however, the thalamofugal pathway but not the tectofugal one shows an asymmetry in its projection pattern whereas cell size asymmetries are not known. In the chickens’ thalamofugal pathway, the left nucleus geniculatus lateralis pars dorsalis (GLd) comprises more projections to right telencephalic visual Wulst than the right GLd to the left visual Wulst. As the ipsilateral GLd-Wulst projections are symmetric between sides, the contralateral projection asymmetry leads to a higher total GLd input on the right visual Wulst (Rogers and Bolden, 1991; Rogers and Deng, 1999). In contrast to the stable tectofugal asymmetries in pigeons (Güntürkün et al., 1998), the lateralization of the chicken’s thalamofugal system only persists for three weeks after hatch (Rogers and Sink, 1988).

### LIGHT-DEPENDENT DEVELOPMENT OF VISUAL ASYMMETRIES

The envirotypic factor light plays an important role for the induction and stabilization of a subset of visual asymmetries in pigeons and chickens. Avian embryos take an asymmetrical position inside the egg, with the right eye pointing towards the semitransparent eggshell. The left eye, however, is occluded by the embryos body (Kuo, 1932). This positioning leads to a stronger light stimulation of the right in comparison to the left eye, which triggers lateralization processes on the anatomical as well as the functional level. In pigeons, this causes an asymmetry in the projections of the tectofugal pathway (Güntürkün et al., 1998) while in chickens projections of the thalamofugal pathway are affected (Rogers and Bolden, 1991; Rogers and Deng, 1999; Koshiba et al., 2003). Dark incubation of eggs prevents establishment of several asymmetries (Rogers and Sink, 1988; Skiba et al., 2002; Manns and Güntürkün, 2003; Freund et al., 2008) and impairs interhemispheric cooperation (Manns and Römling, 2012). Furthermore, in the altricial pigeon, monocular light deprivation during a short plastic period after hatch can strengthen or even alter the direction of visual asymmetries (Manns and Güntürkün, 1999a, b).

In sum, pigeons and chickens develop behavioral as well as anatomical asymmetries depending on the ontogenetic light conditions. But the characteristics of the structural lateralizations differ in at least three major aspects between the two species: (1) the affected pathway (tectofugal in pigeons versus thalamofugal in chickens), (2) the constancy of the lateralization (persistent in pigeons versus transient in chickens), and (3) the hemisphere, which receives stronger bilateral input (left in pigeons versus right in chickens). These differences allow speculating about the causal relations between light-dependent structural and behavioral asymmetries. A closer look at the functional asymmetry pattern of chickens and pigeons suggests that the action of light is more complex as indicated at first glance. Asymmetrical photic stimulation modifies the lateralized interaction of bottom-up and top-down systems that ultimately determine lateralized functional processing.

### INTERRELATIONS BETWEEN LIGHT-DEPENDENT STRUCTURE AND FUNCTION

#### Enhancement of fine-tuned visuomotor circuits within the left hemisphere

Especially left-hemispheric specializations of chickens and pigeons are remarkably similar. Although the behavioral paradigms testing hemispheric asymmetries differ in detail, experiments demonstrate left-hemispheric advantages for visuomotor control that are similar to human left frontal dominances for response inhibition (e.g., Weisbrod et al., 2000; Warren et al., 2013), action planning (Serrien and Sovijärvi-Spapé, 2013), and categorization (e.g., Parrot et al., 1999). The left hemisphere is in charge of the selection of features allowing stimuli to be assigned to discrete categories when discriminating food objects (Mench and Andrew, 1986; Güntürkün and Kesch, 1987; Vallortigara et al., 1996; Rogers, 1997; Rogers et al., 2007) or abstract concepts like humans or painting styles (reviewed in Yamazaki et al., 2007). In pigeons, the superior visual discrimination abilities are related to better left-hemispheric memory capacities when pigeons are required to memorize large numbers of abstract pattern (von Fersen and Güntürkün, 1990) or when they have to perform an object-specific working memory task (Prior and Güntürkün, 2001). Even though the left hemisphere of the chicken brain is not better in using object specific cues in a working memory task (Regolin et al., 2005), it is critically involved in specific forms of quick memory formation like passive avoidance learning (Sandi et al., 1993). In chickens and pigeons, the left hemisphere controls pecking, enabling faster and more accurate responses (Güntürkün, 1985; Güntürkün and Kesch, 1987; Skiba et al., 2002) or inhibiting inappropriate responses (Deng and Rogers, 1997; Rogers et al., 2007).

At least some of the described left-hemispheric dominances emerge in response to asymmetrical photic stimulation during ontogeny. It is well known that sensory experiences have a significant influence over the way the brain is assembled and thus, can functionally impact the way the mature brain works (West and Greenberg, 2011). Transiently enhanced visual input triggers activity-dependent differentiation processes (Manns et al., 2005, 2008; Manns and Güntürkün, 2009; Güntürkün and Manns, 2010) resulting in better fine-tuned visuomotor circuits as demonstrated in numerous plasticity studies (for review e.g., Wong and Ghosh, 2002; Berardi et al., 2003; Espinosa and Stryker, 2012). As a consequence, the left hemisphere of birds is better adjusted to adopt specific visuomotor functions and hence, takes over control. Pre- and posthatch modulations of lateralized visual experience support that the hemisphere that is more strongly activated by light develops a functional dominance (Rogers and Sink, 1988; Manns and Güntürkün, 1999a; Prior et al., 2004a).

Since the emergence of behavioral asymmetries are accompanied by structural left-right differences within the ascending visual pathways (Deng and Rogers, 2000; Manns and Güntürkün, 2009) it is conceivable that they are causally related. A causal relationship would support models proposing that connectivity asymmetries between the hemispheres are critical for cerebral lateralizations since they impact differences in computational principles used by the left and right brain side, which determine their functional properties (Stephan et al., 2007). It is obvious that light input primarily affects the development of ascending visual pathways (Manns and Güntürkün, 2009; Güntürkün and Manns, 2010). Asymmetrical activity-dependent neuronal processes mediate lateralized differentiation of visual neurons leading to asymmetrical neuronal properties that represent the structural correlate of functional lateralizations. In parallel, the ascending systems develop intrinsic functional asymmetries mediating lateralized bottom-up processing (Manns and Güntürkün, 2009; Güntürkün and Manns, 2010). Electrophysiological studies in pigeons have demonstrated more left- than right-rotundal neurons, which respond to contra- as well as ipsilateral visual input (Folta et al., 2004). This is in accordance to the stronger bilateral tectal innervation. Left entopallial neurons are more responsive to visual stimulation and after associative learning they show a higher degree of differentiation between the rewarded and the unrewarded stimulus (Verhaal et al., 2012).

Despite the presence of structural as well as physiological asymmetries in the ascending pathways, the left-hemispheric dominance for visuomotor control cannot simply be based on stronger bottom-up input. A first hint is given by the fact that the visual pathways that show anatomical asymmetries differ between pigeons and chickens (**Figure 1**). Although left-hemispheric development is enhanced in the pigeons’ tectofugal as well as the chickens’ thalamofugal system, stronger bilateral input is guided to the left hemisphere in pigeons but to the right one in chickens. Moreover, only the tectofugal projection asymmetries in pigeons are stable (Güntürkün et al., 1998; Rogers and Deng, 1999) whereas thalamofugal asymmetries in chickens are transient (Deng and Rogers, 2002). Nevertheless, some left hemispheric dominances in hens remain even when projection asymmetries are lost (McKenzie et al., 1998).

This discrepancy can be explained by the critical role of top-down systems onto lateralized visuomotor behavior. Top-down influences arise from the forebrain and exert asymmetrical impact onto visual processing by efferents descending towards the brainstem. Here, they converge onto commissural systems, which regulate lateralization of visuomotor responses in pigeons (Güntürkün and Böhringer, 1987) and chickens (Parsons and Rogers, 1993) and which might be involved in the efficiency of interhemispheric cooperation (Manns and Römling, 2012; Letzner et al., 2014). One source of top-down influences is the hyperpallium or visual Wulst that represents on the one hand the telencephalic target of the thalamofugal pathway (**Figure 1**) but on the other hand a multimodal area reciprocally connected with several telencephalic nuclei (Reiner and Karten, 1983; Shimizu et al., 1995; Deng and Rogers, 2000, cited in Manns et al., 2007). Accordingly, the Wulst is not only a visual structure, but is also involved in higher cognitive functions, playing a role in learning and attentional processes (reviewed in Manns and Güntürkün, 2009). Several studies in pigeons as well as chickens show that the left Wulst exerts a stronger impact onto visuomotor behavior than the right one (Manns and Güntürkün, 2009; Valencia-Alfonso et al., 2009). In pigeons, transient silencing of hyperpallial activity by injections of the sodium channel blocker tetrodotoxin demonstrates that the left Wulst controls tectofugal processing (Folta et al., 2004), modulates access to transfer information (Valencia-Alfonso et al., 2009), and controls motor response in case of conflicting information (Freund et al., 2009.). In chickens, disturbance of neurotransmission by manipulating amino acid pools with telencephalic injections of cycloheximide or glutamate demonstrates that the left hemisphere exerts better inhibitory control on visuomotor behavior than the right one. Only injections into the left but not the right Wulst increase inappropriate pecks onto pebbles in the pebble-grain discrimination task and elevate aggressive and sexual behavior (Rogers and Anson, 1979; Howard et al., 1980; Bullock and Rogers, 1986; Deng and Rogers, 1997, 2002).

It is intriguing that at least some aspects of hyperpallial top-down influences depend on asymmetrical visual experience during embryonic development. Hyperpallial control of categorizing grains as different from pebbles in chickens only emerges in light-stimulated chickens. In dark-incubated birds, treatment of neither the left nor the right Wulst affected performance on the pebble-grain task (Deng and Rogers, 2002). In pigeons, an endogenously present right-hemispheric superiority in accessing visual transfer information is reversed by embryonic light stimulation and it is likely that this effect results from modulations of top-down systems (Letzner et al., 2014).

Although the Wulst represents the telencephalic target of the thalamofugal projection, it is unlikely that the lateralized action of the Wulst depends on structural thalamofugal asymmetries. In pigeons, no thalamofugal projection asymmetries are present at all (Ströckens et al., 2013b). Even in chicks there is dissociation between the development of thalamofugal and behavioral asymmetries. The left-hemispheric dominance in categorizing grains from pebbles depends on the wavelength of the stimulating light and hence, depends on color-coding pathways outside the thalamofugal system. In contrast, thalamofugal projection asymmetries develop independent from wavelength characteristics of the photic stimulus (Rogers and Krebs, 1996).

In sum, we speculate that the emergence of a left-hemispheric dominance in visuomotor control is caused by a transient ontogenetic light trigger independent from the generation of projection asymmetries within ascending visual pathways. A decisive factor is rather the development of lateralized top-down systems. This does not mean that asymmetrical bottom-up projections do not influence lateralized functional processing. In the next paragraph, we will discuss in how far the degree of bilateral ascending input may affect preferential processing strategies and hence, hemispheric dominance in cases of redundant or conflict encoding.

#### Hemispheric-specific processing strategies in analyzing visual stimuli

In principle, environmental stimuli can be analyzed according to different strategies. One is based on a detailed feature analysis attending to local cues. The other one uses global information considering relational cues between stimulus aspects. In principle, both hemispheres can process local as well as global information depending on context and/or -inner states. Nevertheless, several studies in chickens and pigeons demonstrate that the hemispheres differ in their preferential strategies whereby the left hemisphere prefers local, the right one global encoding (Vallortigara and Rogers, 2005; Yamazaki et al., 2007). A conflict can arise when local and global cues provide contradictory information and hence, suggest different response options. In these situations, neuronal mechanisms are required to coordinate a common decision. In many cases, one hemisphere dominates processing and/or behavioral response (Levy and Trevarthen, 1976). Some evidences suggest that pigeons and chickens differ in the dominance pattern for specific functions. Chickens seem to rely more on a right-hemispheric strategy depending on global cues whereas it is the left hemispheres in pigeons that dominates visual processing thereby preferentially encoding local cues (Vallortigara and Rogers, 2005; Daisley et al., 2009; Shimizu et al., 2010; Rosa Salva et al., 2012; Tommasi et al., 2012). A closer look however, indicates that there is some dissociation between hemispheric dominance and processing strategy. This suggests that it is not only an evolutionary based dichotomy in processing style that determines a preferential strategy in analyzing complex visual stimuli. Instead, the lateralized organization of the visual systems may also play a prominent role (Tommasi et al., 2012). We propose that the degree of bilateral input affects the dominant hemisphere and encoding strategy, which are affected by the ontogenetic light conditions in a species-dependent manner.

A first hint comes from social recognition, a cognitive function that is generally assumed to be dominated by right-hemispheric processing (Corballis, 2009; Daisley et al., 2009; Rosa Salva et al., 2012). For example, chicks recognize individual companions and choose to approach cage mates in preference to unfamiliar ones only when using their left eye (Deng and Rogers, 2002). This right-hemispheric dominance is related to the preferential right-hemispheric attention to global feature cues that are used to select mates, identify rivals, locate young, and differentiate members of higher and lower ranks (Rosa Salva et al., 2012). In contrast, pigeons attend to local facial features rather than their configuration when they are required to discriminate between intact faces of conspecifics and globally altered ones in which local features are spatially rearranged (Patton et al., 2010; Shimizu et al., 2010). This strategy fits to a general preference to analyze local elements of visual stimuli and to a general left-hemispheric dominance for categorization (Cavoto and Cook, 2001; Yamazaki et al., 2007; Shimizu et al., 2010). It is not directly tested yet if the preferential encoding of object details is actually related to a left-hemispheric dominance. Verification would indicate a converse dominance pattern for aspects of social recognition in chickens and pigeons that is in correspondence to the hemisphere that receives stronger bilateral visual input.

A second hint is provided by detailed analysis of spatial orientation tasks that indicates dissociation between hemispheric specializations and strategy. Comparable to social recognition, spatial orientation is generally described as a right-hemispheric domain. Accordingly, chickens as well as pigeons place more pecks on objects located within the left visual field indicating a functional dominance of the right hemisphere for visuo-spatial attention comparable to humans (Diekamp et al., 2005; Chiandetti, 2011). But for spatial functions like localization of the own position in space, for orientation, and navigation, more complex spatial processing is required using local, non-geometric as well as global, geometric information about the environment. Several experiments demonstrate that both hemispheres are basically able to encode geometric as well as non-geometric information in natural and semi-natural settings. Nevertheless, orientation behavior under different seeing conditions suggest hemispheric-specific differences in using geometric (global) or non-geometric (local) strategies (Vallortigara and Rogers, 2005; Tommasi et al., 2012). Again, there are evidences for a differential lateralization pattern between chickens and pigeons that mainly arise when spatial cues provide conflicting information.

In a classical study, Tommasi and Vallortigara (2001) trained chicks to locate food buried under sawdust in the center of a square arena providing geometric and/or non-geometric landmark cues. In a conflict situation when landmarks and geometry of the arena point to different localization of food, chicks seeing with the right eye rely on the landmark cues, whereas they consider the geometric information when seeing with the left eye. Thus, chickens demonstrate a clear difference between left- and right-hemispheric search strategies. Moreover, performance under binocular seeing conditions does not differ from the one when seeing with the left eye. This indicates that the right-hemispheric geometric strategy dominates visuospatial orientation (Tommasi and Vallortigara, 2001). Unilateral hippocampal lesions confirmed this pattern (Tommasi et al., 2003). A completely different pattern was detected in pigeons that were trained in a very similar task (Wilzeck et al., 2009). Although monocular tests confirm that each brain hemisphere consider geometric as well as landmark information, both hemispheres encode landmark information more heavily than geometric one in conflict situations. Only when using both eyes, pigeons rely preferentially on geometric cues. Thus, in contrast to chickens, pigeons do not demonstrate an asymmetry in monocular search strategy; they rather display a preferential use of a local encoding strategy that is not bound to one hemisphere.

A similar species difference in hemispheric-specific contributions to search strategies could be detected in spatial working memory tasks combining object- and position-specific information. Chicks show a right-hemispheric dominance for locating a target on the basis of position-dependent cues but participation of both hemispheres is required for locating a target on the basis of object-specific cues. When object and positional cues provide contradictory information, the right hemisphere preferentially attends to position-specific, geometric cues, whereas the left hemisphere tends to attend to object-specific features. When seeing with both eyes, chickens attend to geometric cues supporting the dominance of the right-hemispheric strategy (Regolin et al., 2005). A similar working memory task with pigeons shows that the left hemisphere is dominant in processing object-specific/local information while both hemispheres encode global geometric information to an equal degree (Prior and Güntürkün, 2001). Thus, in contrast to chicken, the left hemisphere of pigeons is not only specialized for local visual analysis but also attends to global features. This is supported by hippocampal lesion studies demonstrating that the left hippocampus is critically involved in the representation of a goal when geometric encoding is required (Nardi and Bingman, 2007). Accordingly, the left hemisphere plays generally a more important role in natural homing behavior (Ulrich et al., 1999; Prior et al., 2004b).

In sum, spatial reference and working memory tasks demonstrate a clearly lateralized use of spatial information in chickens: the left hemisphere encodes local non-geometric information and the right one relies on global, geometric cues. This pattern supports an evolutionary conserved dichotomy. Moreover, preferential encoding of geometric information under binocular seeing conditions demonstrates the dominance of the right-hemispheric global strategy. In pigeons, however, there is evidence for a dominance of the left hemisphere in spatial orientation tasks whereby it does not only use local but also global cues. An explanation for this differential pattern might be related to the differential organization of the ascending visual pathways. The right-hemispheric dominance in chickens is in accordance with the stronger bilateral input and hence, right-hemispheric activation even under binocular seeing conditions. In contrast, the stronger innervation of the left hemisphere in pigeons leads to enhanced left-hemispheric activation. Accordingly, even when seeing with the left eye, the left hemisphere is strongly activated and dominates visual analysis as indicated by the preferential encoding of local feature cues. On the other hand, since the left hemisphere is also able to encode global information, suitable tasks demonstrate a left-hemispheric dominance independent from available visual cues. Dominance may result from a more complete representation and/or simply enhanced hemispheric activation due to a stronger bilateral input. The contribution of different visual pathways indicate some species-dependent differences; but since the degree of bilateral input to the hemispheres is controlled by the ontogenetic light conditions, the differential hemispheric-specific encoding pattern further supports the critical role of environmental factors.

## CONCLUSION: SIMILAR BUT DIFFERENT – HOW ONE ENVIROTYPIC FACTOR AFFECTS THE INTERACTION OF BOTTOM-UP AND TOP-DOWN SYSTEMS

A close comparison of the two most intensively studied avian models – chickens and pigeons- sheds light onto three aspects of cerebral lateralization: (1) it exemplifies the critical impact of an envirotypic factor for the generation of a lateralized neuronal system whose action is superimposed on endogenous asymmetries. (2) It indicates dissociation between structural and functional asymmetries that are (3) related to an intimate interaction of bottom-up and top-down systems in a species-dependent manner – an interaction that is much more complex than originally assumed (e.g., Gilbert and Li, 2013).

In chickens as well as pigeons, asymmetrical visual light experience during embryonic development leads to structural and functional lateralizations of their visual systems. A left-hemispheric dominance in visuomotor control is induced by shortly enhanced photic stimulation and is accompanied by the emergence of projection asymmetries in the ascending pathways. Which visual pathway develops structural asymmetries seems to depend on species-dependent differences in the ontogenetic susceptibility to light stimulation (Ströckens et al., 2013b); however, they are not a prerequisite for the generation of hemispheric dominance.

Ultimate consequences of biased visual experience may be established at forebrain level from where lateralized top-down systems control visual processing. Top-down asymmetries develop as secondary consequences of asymmetrical visual stimulation, presumably during posthatch stabilization of induced asymmetries involving negative feedback loops, which preserve asymmetries even in the absence of lateralized input (Manns, 2006; Manns and Güntürkün, 2009). Thereby they may differentiate own microstructural asymmetries but, known as up to now, no asymmetries in efferent projections (Manns et al., 2007). Once established, higher lateralized (top-down) systems are not necessarily longer dependent on asymmetrical bottom-up input. They can exert their action on visual processing presumably by mesencephalic commissural systems onto which ascending and descending visual pathways converge (Manns and Güntürkün, 2009; Güntürkün and Manns, 2010). In turn, these commissural systems regulate lateralization of visuomotor control in pigeons (Güntürkün and Böhringer, 1987) and chickens (Parsons and Rogers, 1993) and might be involved in the efficiency of interhemispheric cooperation (Manns and Römling, 2012; Letzner et al., 2014).

This critical impact of lateralized top-down processes in no way means, that stable bottom-up asymmetries do not affect hemispheric dominances. On the one hand, asymmetrical projections may result in asymmetrical salience of stimuli represented within the left and right hemisphere eventually triggering different processing strategies. On the other hand, asymmetrical innervation may cause enhanced activation of the hemisphere that receives stronger bottom-up input. As a consequence, this hemisphere is quicker in response generation or may recruit more attentional resources and hence, dominates visuomotor processing as a result of a “horse race” between the hemispheres (e.g., Corballis, 1998). This idea is supported by hints for left-hemispheric metacontrol in pigeons (Adam and Güntürkün, 2009; Freund et al., 2009). The absence of similar metacontrol in chickens would suggest that permanent asymmetrical bottom-up systems are critical for hemispheric dominances.

The critical role of lateralized bottom-up systems as indicated by the degree of bilateral ascending projections may also tackle another basic aspect of hemispheric-specific processing. It is intriguing that although left-hemispheric development is enhanced in the pigeons’ tectofugal as well as the chicken’s thalamofugal system, stronger bilateral input is guided to the left hemisphere in pigeons but to the right one in chickens. This may lead to a differential degree of activation and may influence the balance of left- and right-hemispheric processing. Although both hemispheres can encode local as well as global feature cues, the hemispheres differ in their preferential encoding strategies. This lateralization seems to have some phylogenetic foundation (Vallortigara and Rogers, 2005; McGilchrist, 2010; Concha et al., 2012) but might be affected by ontogenetic experiences. Comparing the lateralization patterns of pigeons and chickens, we propose that the degree of bilateral visual input influences the use of encoding strategies, which therefore depends on asymmetrical photic stimulation. This hypothesis still has to be tested in animals with different ontogenetic light experiences. These studies will provide important clues for a deeper understanding of the experience-dependent interplay between bottom-up and top-down processing that are superimposed by species-dependent endogenous asymmetries.

## Conflict of Interest Statement

The authors declare that the research was conducted in the absence of any commercial or financial relationships that could be construed as a potential conflict of interest.
